# Synaptic Connectivity in Medium Spiny Neurons of the Nucleus Accumbens: A Sex-Dependent Mechanism Underlying Apathy in the HIV-1 Transgenic Rat

**DOI:** 10.3389/fnbeh.2018.00285

**Published:** 2018-11-22

**Authors:** Kristen A. McLaurin, Anna K. Cook, Hailong Li, Alexis F. League, Charles F. Mactutus, Rosemarie M. Booze

**Affiliations:** Department of Psychology, Program in Behavioral Neuroscience, University of South Carolina, Columbia, SC, United States

**Keywords:** HIV-1 transgenic rat, biological sex, apathy, medium spiny neurons, dopamine, diagnostic classification, discriminant function analysis

## Abstract

Frontal-subcortical circuit dysfunction is commonly associated with apathy, a neuropsychiatric sequelae of human immunodeficiency virus type-1 (HIV-1). Behavioral and neurochemical indices of apathy in the nucleus accumbens (NAc), a key brain region involved in frontal-subcortical circuitry, are influenced by the factor of biological sex. Despite evidence of sex differences in HIV-1, the effect of biological sex on medium spiny neurons (MSNs), which are central integrators of frontal-subcortical input, has not been systematically evaluated. In the present study, a DiOlistic labeling technique was used to investigate the role of long-term HIV-1 viral protein exposure, the factor of biological sex, and their possible interaction, on synaptic dysfunction in MSNs of the NAc in the HIV-1 transgenic (Tg) rat. HIV-1 Tg rats, independent of biological sex, displayed profound alterations in synaptic connectivity, evidenced by a prominent shift in the distribution of dendritic spines. Female HIV-1 Tg rats, but not male HIV-1 Tg rats, exhibited alterations in dendritic branching and neuronal arbor complexity relative to control animals, supporting an alteration in glutamate neurotransmission. Morphologically, HIV-1 Tg male, but not female HIV-1 Tg rats, displayed a population shift towards decreased dendritic spine volume, suggesting decreased synaptic area, relative to control animals. Synaptic dysfunction accurately identified presence of the HIV-1 transgene, dependent upon biological sex, with at least 80% accuracy (i.e., Male: 80%; Female: 90%). Collectively, these results support a primary alteration in circuit connectivity, the mechanism of which is dependent upon biological sex. Understanding the effect of biological sex on the underlying neural mechanism for HIV-1 associated apathy is vital for the development of sex-based therapeutics and cure strategies.

## Introduction

Apathy, one of the neuropsychiatric sequelae of human immunodeficiency virus type 1 (HIV-1), is operationally defined as the quantitative reduction of self-generated voluntary and purposeful (goal-directed) behaviors (Levy and Dubois, 2006). Despite treatment with combination antiretroviral therapy (cART), approximately 30%–60% of HIV-1 seropositive individuals exhibit motivational alterations (Kamat et al., 2012; Marquine et al., 2014); alterations which are associated with functional consequences, including difficulties conducting instrumental activities of daily living (e.g., Kamat et al., 2012, 2013, 2016; Shapiro et al., 2013) and decreased medication adherence (Panos et al., 2014). Given the prevalence of apathy in HIV-1 seropositive individuals, there is a critical need to elucidate the structural loci for the actions of HIV-1 viral proteins on goal-directed behaviors.

Frontal-subcortical circuit dysfunction is commonly associated with impaired executive functioning (including alterations in sustained attention and memory) and apathy (review, Bonelli and Cummings, 2007). Broadly, the frontal-subcortical circuit, originally proposed by Alexander and colleagues (Alexander et al., 1986; Alexander and Crutcher, 1990; Alexander, 1994), includes five parallel segregated circuits linking the basal ganglia and prefrontal cortex (PFC). However, the anterior cingulate circuit, considered one of the behaviorally relevant frontal-subcortical circuits, is more directly related to motivational alterations (Bonelli and Cummings, 2007). Neurons from the anterior cingulate cortex (ACC) send projections to the ventral striatum, including the nucleus accumbens (NAc; Selemon and Goldman-Rakic, 1985), which subsequently innervate the globus pallidus interna, ventral pallidum and rostrodorsal substantia nigra (Critchley, 2005).

Aside from alterations in neuroanatomical circuitry, dopaminergic system dysfunction may also play a role in apathy (review, Chong and Husain, 2016). Specifically, key brain regions within the frontal-subcortical circuit, including the ACC and ventral striatum, receive dopaminergic projections from the ventral tegmental area (VTA). The direct relationship between dopamine (DA) system dysfunction and apathy has been investigated by treating non-human primates with the dopaminergic neurotoxin 1-methyl-4-phenyl-1,2,3,6-tetrahydropyridine (MPTP; Brown et al., 2012; Tian et al., 2015). Dose-dependent increases in apathy, assessed using an animal’s willingness to attempt goal-directed behaviors, were observed following infusions of MPTP (Brown et al., 2012). More critically, apathetic behaviors were significantly inversely correlated (i.e., *R*^2^ > 0.51) with dopaminergic terminal integrity in the NAc core subregion (Brown et al., 2012), as well as dopaminergic projections from the VTA to cortical regions, including the dorsolateral PFC and ventromedial PFC (Tian et al., 2015). The effective treatment of apathy with dopaminergic agonists, including methylphenidate (e.g., Padala et al., 2018), ropinirole (e.g., Kohno et al., 2010; Blundo and Gerace, 2015), and piribedil (Thobois et al., 2013) provides additional evidence for the role of DA system dysfunction in the etiology of the disorder.

Gamma-aminobutyric acid (GABA) medium spiny neurons (MSNs) are the major inhibitory projection neurons in the NAc, comprising approximately 95% of the cells within the region (Kemp and Powell, 1971). Nearly every MSN is innervated by dopaminergic axons via afferents from the VTA (Yao et al., 2008). The PFC, thalamus, and hippocampus (Harris and Stevens, 1989), as well as the amygdala (Bredt and Nicoll, 2003) also send glutamatergic inputs to MSNs of the NAc. Morphologically, MSNs are characterized by high densities of dendritic spines (Cheng et al., 1997) which serve as the main postsynaptic compartment of excitatory synapses (Harris and Kater, 1994), and may reflect neuronal processing capacity (Mancuso et al., 2012). Functionally, many motivational (e.g., Ostlund et al., 2014) and goal-directed behaviors (e.g., Shen et al., 2009) are influenced by MSN function.

Prominent sex differences are commonly observed in preclinical studies of apathy (e.g., Roberts et al., 1989; Richardson and Roberts, 1996; Lynch and Taylor, 2005; Ramôa et al., 2013) and may be due, at least in part, to selective neuroanatomical differences in MSNs of the NAc. Specifically, an increased spine density and higher proportion of large spines are observed in female animals relative to males; effect that are primarily observed on the most distal dendritic branches and are stronger in the NAc core subregion (Forlano and Woolley, 2010; Wissman et al., 2011). Neurochemically, female animals displayed significantly greater miniature excitatory post-synaptic current (mEPSC) frequency, suggesting greater surface expression of AMPARs on MSNs (Wissman et al., 2011); an effect which may be mediated by ovarian hormones, including estrogen (Cao et al., 2016). However, no significant sex differences were observed in mEPSC amplitude (Wissman et al., 2011), or MSN action potential properties (Cao et al., 2016). Although depression and apathy are distinct psychopathological conditions, it is noteworthy that sex differences have also been observed in NAc gene expression (Hodes et al., 2015) and glutamatergic inputs into MSN of the NAc (Brancato et al., 2017) using a rodent model of depression. Collectively, results suggest that sex differences in neuroanatomical afferents onto MSNs may mechanistically underlie differences in circuitry function (Cao et al., 2018).

Most notably, damage to frontal-subcortical circuitry (e.g., Cole et al., 2007), DA system dysfunction (e.g., Kumar et al., 2009, 2011) and prominent sex differences (e.g., Hestad et al., 2012; Royal et al., 2016; Maki et al., 2018) have been observed in HIV-1 seropositive individuals; observations which were translationally modeled in the HIV-1 transgenic (Tg) rat (e.g., Vigorito et al., 2007; Moran et al., 2014; Javadi-Paydar et al., 2017; McLaurin et al., 2017; Bertrand et al., 2018). Specifically, the HIV-1 Tg rat, originally reported by Reid et al. (2001), displays profound neurocognitive alterations in tasks tapping frontal-subcortical circuitry (e.g., signal detection: Moran et al., 2014; McLaurin et al., 2017; Morris Water Maze: Vigorito et al., 2007), as well as motivational dysregulation (Bertrand et al., 2018). Additionally, HIV-1 Tg female animals exhibited increased DA reuptake time (Javadi-Paydar et al., 2017) and profound dysregulation of DA in the striatum (Lee et al., 2014; Bertrand et al., 2018). Morphological alterations in MSNs in the NAc core subregion, previously reported in young female HIV-1 Tg rats, provide additional evidence for DA system dysfunction in HIV-1 (Roscoe et al., 2014; Javadi-Paydar et al., 2017). To date, however, the effect of biological sex on MSNs, which are central integrators of frontal-subcortical input, has not been systematically evaluated.

Investigating sex differences in the neural mechanisms underlying HIV-1 associated apathy is vital, as women are inadequately represented in both clinical and preclinical studies (Maki and Martin-Thormeyer, 2009). Women represent approximately 51% of HIV-1 seropositive individuals (UNAIDS, 2016) and are more vulnerable to neurocognitive dysfunction (Royal et al., 2016; Maki et al., 2018) relative to HIV-1 seropositive men. The interaction between HIV-1 and biological sex on neurobehavioral alterations, including apathy, however, has not yet been systematically evaluated.

Thus, in the present study, synaptic dysfunction was assessed using a DiOlistic labeling technique in MSNs in the NAc core subregion of male and female HIV-1 Tg rats at an advanced age. Specifically, alterations in neuronal arbor complexity and the distribution of dendritic spines, an assessment of synaptic connectivity, were assessed using Sholl analyses (Sholl, 1953). Alterations in dendritic branching were examined using a centrifugal branch ordering method. Population shifts in dendritic spine morphology were subsequently examined with a primary focus on dendritic spine volume. Understanding the effect of biological sex on the underlying neural mechanism for HIV-1 associated apathy is vital for the development of sex-based therapeutics and cure strategies.

## Materials and Methods

### Animals

Intact male and female Fischer (F344/N; Harlan Laboratories Inc., Indianapolis, IN, USA) HIV-1 Tg (*N* = 20 litters) and control (*N* = 17 litters) rats arrived at the animal vivarium, housed with their biological dam, between PD 7 and PD 9 across 12 months. At weaning, which occurred at approximately PD 21, animals were sampled from each litter (HIV-1 Tg: male, *n* = 37, female, *n* = 33; Control: male *n*=34; female, *n* = 33) and pair- or group-housed with animals of the same sex for the duration of experimentation.

HIV-1 Tg and control animals were housed in AAALAC-accredited facilities using the guidelines established in the Guide for the Care and Use of Laboratory Animals of the National Institutes of Health. The targeted environmental conditions for the animal vivarium were 21° ± 2°C, 50% ± 10% relative humidity and a 12-h light:12-h dark cycle with lights on at 07:00 h (EST). The project protocol was approved under Federal Assurance (#D16-00028) by the Institutional Animal Care and Use Committee (IACUC) at the University of South Carolina.

Animals were placed on food restriction (Pro-Lab Rat, Mouse, Hamster Chow #3000) at approximately PD 60 to maintain 85% body weight due to a concurrently conducted signal detection operant task. After animals successfully acquired the signal detection task (PD 100–PD 277), rodent food was again provided *ad libitum* for the duration of the study. Water was provided *ad libitum* to all animals throughout the duration of the study.

Assessments of cross-modal prepulse inhibition (PPI), gap prepulse inhibition (gap-PPI), and locomotor activity were conducted at approximately 18 months of age. Following a history of neurocognitive testing (i.e., sustained attention and reversal via a signal detection operant task, PPI, gap-PPI, and locomotor activity), HIV-1 Tg animals were sacrificed at approximately 20 months of age to assess synaptic dysfunction. Some animals were euthanized prior to 20 months of age due to significant health issues.

Investigation of the effects of long-term HIV-1 viral protein exposure in aged (i.e., 20 months of age) animals is clinically relevant given the dramatic increase in life expectancy following the advent of cART (e.g., Romley et al., 2014; Teeraananchai et al., 2017). In the United States, older individuals (>50 years of age) account for approximately 47% of all HIV-1 seropositive individuals (Centers for Disease Control and Prevention, 2018). More notably, however, by 2030, prediction models suggest the prevalence of older individuals living with HIV-1 will reach approximately 73% (Smit et al., 2015).

### Synaptic Dysfunction

#### Preparation of Tissue

Sevoflurane (Abbot Laboratories, North Chicago, IL, USA) was used to deeply anesthetize animals prior to transcardial perfusion, conducted using methodology adapted from Roscoe et al. (2014). In brief, transcardial perfusion was performed using 100 mL of 100 mM PBS wash followed by 100–150 mL of 4% paraformaldehyde buffered in PBS (Sigmal-Aldrich, St. Louis, MO, USA). Following perfusion, brains were dissected and post-fixed in 4% paraformaldehyde for 10 min. Coronal slices were cut 1 mm thick using a rat brain matrix (ASI Instruments, Warren, MI, USA), washed 3× in PBS, notched for orientation, and placed in a tissue cell culture plate (24-well plate; Corning, Tewksbury, MA, USA).

#### DiOlistic Labeling

MSNs from the NAc core subregion were visualized using a DiOlisitc labeling technique; a technique described comprehensively by Seabold et al. (2010) in a methodology protocol.

Methodology for the preparation of DiOlistic cartridges, preparation of Tefzel tubing, and DiOlistic labeling is described in detailed by McLaurin et al. (2018b). In brief, 170 mg of tungsten beads (Bio-Rad, Hercules, CA, USA) were dissolved in 99.5% pure methylene chloride (Sigma-Aldrich, St. Louis, MO, USA) to prepare DiOlistic cartridges. The bead solution (100 μl) was placed on a glass slide and topped with 150 μl crystallized DiI (6 mg; Invitrogen, Carlsbad, CA, USA) dissolved in methylene chloride. After the dye/bead mixture was allowed to air dry, it was collected with wax-coated weigh paper, and transferred to a 15 ml conical tube (BD Falcon, San Jose, CA, USA) with 3 ml ddH_2_O and sonicated for 10–15 min. The dye/bead mixture was slowly drawn into Tefzel tubing (IDEX Health Sciences, Oak Harbor, WA, USA) and placed in the tubing prep station (Bio-Rad) for 5 min. Using nitrogen gas, the tubing was fully dried and cut into 13 mm segments.

The Helios gene gun (Bio-Rad, Hercules, CA, USA), loaded with previously prepared DiOlistic cartridges, was utilized for DiOlistic labeling. Helium gas flow was adjusted to 80 PSI for ballistic delivery through 3 μm pore filter paper and approximately 2.5 cm away from the sample. For dye diffusion, sections were washed 3× in PBS and stored overnight at 4°C. Tissue sections were mounted using Pro-Long Gold Antifade (Invitrogen, Carlsbad, CA, USA), cover slipped (#1 cover slip; Thermo Fisher Scientific, Waltham, MA, USA) and stored in the dark at 4°C.

#### Medium Spiny Neuron Dendritic Analysis and Spine Quantification

MSNs from the NAc core subregion, located approximately 2.76 mm anterior to Bregma (Paxinos and Watson, 2014), were analyzed. Z-stack images of three to four MSNs were obtained from each animal (Control: *N* = 17 litters, male, *n* = 31, female, *n* = 30; HIV-1 Tg: *N* = 20 litters, male, *n* = 35, female, *n* = 33) using a Nikon TE-2000E confocal microscope utilizing Nikon’s EZ-C1 software (version 3.81b). Magnification was set at 60 × (n.a. = 1.4) and Z-plane intervals of 0.15 μm (pinhole size 30 μm; back-projected pinhole radius 167 nm) were used. DiI fluorophore excitation was accomplished using a green helium-neon (HeNe) laser with an emission of 533 nm.

One neuron from each animal was chosen for the analysis of spine parameters, Sholl analysis and dendritic branching based on several selection criteria (e.g., continuous dendritic staining, low background/dye clusters, minimal diffusion of the DiI dye into the extracellular space). Only neurons meeting the selection criteria were included in the analysis, yielding Control, *N* = 17 litters, male, *n* = 28, female, *n* = 26, and HIV-1 Tg *N* = 20 litters, male, *n* = 29, female, *n* = 32. Following selection, neurons were reconstructed and traced using the AutoNeuron extension module within Neurolucida 360 (MicroBrightfield, Williston, VT, USA). Subsequently, dendritic spines were detected and spine parameters were quantified by utilizing the Neurolucida 360 AutoSpine extension module.

#### Spine Parameters

An algorithm in Neurolucida 360 (Rodriguez et al., 2008) was used for observer-assisted automatic classification of dendritic spines (i.e., thin, mushroom, stubby). Sholl analyses were conducted using Neurolucida 360 to evaluate neuronal arbor complexity (i.e., the number of intersections at each successive radii) and dendritic spine connectivity (i.e., the number of dendritic spines between each successive radii). The radius interval for the Sholl analysis was set to 10 μm. Additionally, the number of segments at each branch order, an assessment of dendritic branching complexity, was examined for branch orders 1 through 10, established using a centrifugal branch ordering method. Dendritic spine morphology was assessed using three parameters, including backbone length (μm), head diameter (μm) and volume (μm^3^). Given that dendritic spine volume is dependent upon both dendritic spine backbone length and dendritic spine head diameter, only dendritic spine volume is presented. Parameters for volume were established using well-accepted previously published results, such that dendritic spines with a volume of 0.05–0.5 μm^3^ were included in the analysis (Hering and Sheng, 2001).

### Integrity of Sensory System Function: Prepulse Inhibition

#### Apparatus

The startle platform (SR-Lab Startle Reflex System, San Diego Instruments, Inc., San Diego, CA, USA) was enclosed within an isolation cabinet (external dimensions: 10 cm-thick, double-walled, 81 × 81 × 116-cm; Industrial Acoustic Company, INC., Bronx, NY, USA), instead of the 1.9 cm thick ABS plastic or laminate cabinets offered with this system. Enclosure within the isolation cabinet provided 30 dB(A) of sound attenuation relative to the external environment. Throughout the testing sessions, background noise of 70 dB(A) was delivered. A high-frequency loudspeaker of the SR-Lab system (model#40-1278B, Radio Shack, Fort Worth, TX, USA), used to present all auditory prepulse and startle stimuli, was mounted 30 cm above the Plexiglas animal test cylinder and calibrated using a sound level meter (model #2203, Bruël and Kjaer, Norcross, GA, USA). A white LED light (22 lux; Light meter model #840006, Sper Scientific, Ltd, Scottsdale, AZ, USA), used for the presentation of all visual prepulse stimuli, was affixed on the wall in front of the test cylinder. A piezoelectric accelerometer integral to the bottom of the cylinder converted the deflection of the test cylinder, resulting from the animal’s response to the auditory stimulus, into analog signals. Response signals were digitized (12 bit A to D, recorded at a rate of 2,000 samples/s) and saved to a hard disk. Response sensitivities were calibrated using the SR-LAB Startle Calibration System.

#### Cross-Modal Prepulse Inhibition

At approximately 18 months of age (i.e., PD 540), HIV-1 Tg and control animals were assessed using a cross-modal PPI experimental paradigm to assess sensory system functioning (HIV-1 Tg: *N* = 20 litters, male, *n* = 27, female, *n* = 30; Control: *N* = 17 litters, male *n*=29; female, *n* = 31). Cross-modal PPI was conducted similar to our prior publications (e.g., Moran et al., 2013a). In brief, the cross-modal PPI testing session, conducted in the dark, was approximately 30 min long and began with a 5 min acclimation period. Subsequently, six pulse-only auditory startle response (ASR) trials, used for habituation, were presented with a fixed 10 s intertrial interval (ITI). Seventy-two testing trials, including an equal number of auditory and visual prepulse trials, were presented in a counterbalanced order (i.e., ABBA) to control for order-effects. A Latin-square experimental design with 6-trial blocks was used for the presentation of all ISIs (0, 30, 50, 100, 200, 4,000 ms). All testing trials had a variable ITI (15–25 s). The 0 and 4,000 ms ISIs were considered control trials, providing a reference ASR within the cross-modal PPI assessment. All prepulse stimuli (auditory: 85 dB(A) white noise stimulus; visual: 22 lux)) and the auditory startle stimulus (100 dB(A)) had a duration of 20 ms. Peak ASR amplitude values were collected for further analysis.

#### Gap Prepulse Inhibition

Gap-PPI of the ASR was assessed after cross-modal PPI and locomotor activity at approximately 18 months of age (i.e., PD 540; HIV-1 Tg: *N* = 20 litters, male, *n* = 27, female, *n* = 30; Control: *N* = 17 litters, male *n* = 28; female, *n* = 31). Methodology for conducting gap-PPI is similar to our prior publication (McLaurin et al., 2016). In brief, a 20 min test session, beginning with a 5 min acclimation period, was conducted in the dark with 70 dB(A) background white noise. Six pulse-only ASR trials, including a fixed 10 s ITI, were used for habituation. Thirty-six testing trials were presented in 6-trial blocks interdigitated using a Latin Square experimental design with a variable ITI (15–25 s). ISIs of 30, 50, 100, and 200 ms included a 20 ms gap in white noise preceding the auditory startle stimulus (100 dB(A) intensity with a 20 ms duration). To provide a reference ASR within the gap-PPI assessment, the session included two control trials with ISIs of 0 and 4,000 ms. Analyses were conducted on the peak ASR amplitude values.

### Integrity of Gross-Motoric System Function: Locomotor Activity

#### Apparatus

Perspex inserts were used to convert square (40 × 40 cm) activity monitors (Hamilton Kinder, San Diego Instruments, San Diego, CA, USA) into round (~40 cm diameter) compartments for the assessment of locomotor activity. Free movement was detected using infrared photocell (32 emitter/detector pairs) interruptions. The sensitivity of the photocells was tuned by the manufacturer to maintain their sensitivity with the additional layer of perspex.

#### Procedure

Locomotor activity was assessed at approximately 18 months of age (i.e., PD 540) as an assessment of gross motoric system function (HIV-1 Tg: *N* = 20 litters, male, *n* = 26, female, *n* = 31; Control: *N* = 17 litters, male *n*=29; female, *n* = 31). Sixty minute test sessions were conducted in an isolated room under dim lighting conditions (<10 lux) between 7:00 h and 12:00 h (EST).

### Statistical Analysis

Data were analyzed using analysis of variance (ANOVA) and regression techniques (SAS/STAT Software 9.4, SAS Institute, Inc., Cary, NC, USA; GraphPad Software, Inc., La Jolla, CA, USA; SPSS Statistics 24, IBM Corp., Somer, NY, USA). GraphPad Prism 5 was utilized to create all figures (GraphPad Software, Inc., La Jolla, CA, USA). Given the nested experimental design, which leads to a violation of the ANOVA assumption of independence, individual observations were analyzed using litter means and standard errors, dependent upon biological sex (Denenberg, 1984; Wears, 2002). An alpha criterion of *p* ≤ 0.05 was used for the evaluation of all statistical tests.

Dendritic spine connectivity was assessed by examining the number of dendritic spines, dependent upon spine type (i.e., thin, stubby, mushroom), between each radii. Dendritic branching complexity was examined by analyzing the number of dendrites at each branch order. Dendritic spine volume served as a measurement of dendritic spine morphology. A generalized linear mixed effects model with either a Poisson distribution or a normal distribution and an unstructured covariance pattern was conducted using PROC GLIMMIX (SAS/STAT Software 9.4, SAS Institute, Inc., Cary, NC, USA) for dendritic spine connectivity, dendritic branching complexity, and dendritic spine volume. Specifically for the assessment of dendritic spine connectivity, a normal distribution was utilized for the overall model, as well as for the follow-up assessment of thin spines. However, a Poisson distribution was utilized for follow-up assessments of stubby spines and mushroom spines. Analyses were conducted on the number of dendritic spines between each successive radii or the number of dendritic spines within each bin. Spine Type, radii, and bin served as within-subjects factors, as appropriate, while genotype (HIV-1 Tg vs. Control) and biological sex (Male vs. Female) served as between-subjects factors. A method equivalent to maximum likelihood estimation was utilized for dendritic spine connectivity, while a maximum likelihood estimation based on adaptive quadrature was used for dendritic branching complexity and dendritic spine volume.

Neuronal arbor complexity, assessed using the number of intersections at successive radii (i.e., Sholl analysis), was analyzed using a mixed-design ANOVA and restricted maximum likelihood estimation model parameters (SAS/STAT Software 9.4, SAS Institute, Inc., Cary, NC, USA). An autoregressive covariance structure was utilized due to the analysis of sequential measurements at successive radii (Wilson et al., 2017). The model allowed for both a random intercept, as well as a random slope (i.e., Radius). Genotype (HIV-1 Tg vs. Control) and biological sex (Male vs. Female) served as the between-subjects factors.

The utility of dendritic spine alterations to correctly classify animals based on their genotype was assessed using a discriminant function analysis (DFA; SPSS Statistics 24, IBM Corp., Somer, NY, USA). Given the sex differences observed in dendritic spine alterations, analyses of each sex were conducted independently.

The functional health of the HIV-1 Tg rat was assessed using cross-modal PPI, gap-PPI and locomotor activity. Analyses utilized a mixed-design ANOVA with restricted maximum likelihood estimation model parameters (SAS/STAT Software 9.4, SAS Institute, Inc., Cary, NC, USA). A compound symmetry (cross-modal PPI, gap-PPI) or unstructured (locomotor activity) covariance structure was utilized. ISI and trial served as the within-subjects factors, as appropriate, while genotype (HIV-1 Tg vs. Control) and biological sex (Male vs. Female) served as the between-subjects factors.

## Results

### HIV-1 Tg Animals, Independent of Biological Sex, Displayed a Profound Alteration in Dendritic Spine Distribution, Supporting an Alteration in Synaptic Connectivity

**Figure 1:**
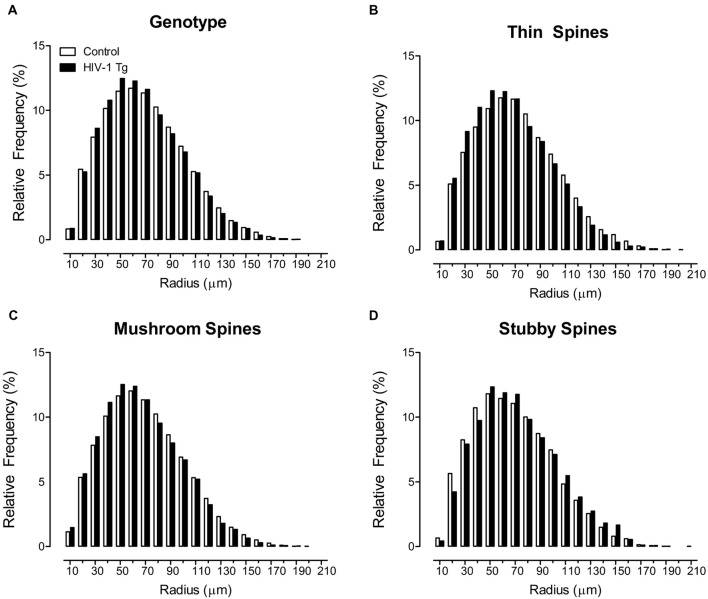
Profound shifts in the distribution of dendritic spines on a neuron are illustrated as a function of genotype. A prominent shift in the overall distribution of 500,000+ dendritic spines, collapsed across spine type **(A)**, is observed in human immunodeficiency virus type-1 (HIV-1) transgenic (Tg) animals relative to control animals. Specifically, HIV-1 Tg animals exhibited an increased relative frequency of dendritic spines on branches more proximal to the soma; an effect that was dependent upon spine type. HIV-1 Tg animals, relative to controls, displayed an increased relative frequency of thin **(B)** and mushroom **(C)** spines on more proximal branches. In sharp contrast, HIV-1 Tg animals exhibited an increased relative frequency of stubby spines **(D)** on more distal branches relative to control animals.

Sholl analyses were used to determine where dendritic spines were located on the neuron, assessed using spine type (i.e., Thin Spines, Stubby Spines, Mushroom Spines), determined using an observer-assisted automatic classification system in Neurolucida 360, genotype (i.e., HIV-1 Tg vs. Control), biological sex (i.e., Male vs. Female) and radii as factors (Figure 1). HIV -1 Tg animals, independent of biological sex, exhibited a prominent distributional shift, with an increased frequency of dendritic spines closer to the soma (Figure 1A), confirmed via a significant radii × genotype interaction (*F*_(1,7165)_ = 6.9, *p* ≤ 0.009).

Most notably, the distributional shift in the where dendritic spines were located on the neuron was dependent upon both the factor of biological sex and spine type (radii × genotype × sex × spine type interaction (*F*_(2,7165)_ = 3.2, *p* ≤ 0.042); Supplementary Figure S1). Complementary analyses were conducted for each spine type to determine the locus of the interaction. Specifically, for thin (Figure 1B) and mushroom spines (Figure 1C), HIV-1 Tg animals displayed an increased frequency of dendritic spines on more proximal branches, relative to control animals (Thin: radii × genotype interaction (*F*_(1,2351)_ = 8.1, *p* ≤ 0.004); Mushroom: radii × genotype interaction (*F*_(1,2351)_ = 60.6, *p* ≤ 0.001), the magnitude of which is dependent upon biological sex (Thin: radii × genotype × sex interaction (*F*_(1,2351)_ = 4.5, *p* ≤ 0.034); Mushroom: radii × genotype × sex interaction (*F*_(1,2351)_ = 5.9, *p* ≤ 0.015)). In sharp contrast, for stubby spines, HIV-1 Tg animals, independent of biological sex, exhibited a distributional shift with an increased frequency of dendritic spines on more distal branches (Figure 1D; radii × genotype interaction (*F*_(1,2351)_ = 56.1, *p* ≤ 0.001)).

### Alterations in Neuronal Morphology, Evidenced by Profound Alterations in MSN Dendritic Branching and Dendritic Arbor Complexity, Were Observed in Female, but Not Male, HIV-1 Tg Animals

#### Branch Order

Neurolucida 360 utilized a centrifugal branch ordering method to automatically assign each dendrite with a branch order by counting the number of segments traversed. Presence of the HIV-1 transgene had a profound effect on dendritic branching in MSNs of the NAc core subregion, dependent upon the factor of biological sex, evidenced by a significant branch order × sex × genotype interaction (*F*_(1,1086)_ = 12.1, *p* ≤ 0.001).

**Figure 2:**
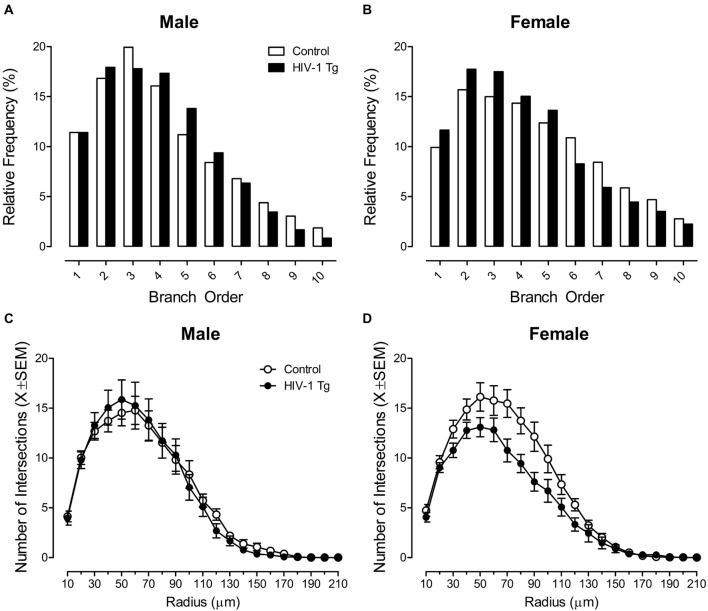
Presence of the HIV-1 transgene had a profound influence on neuronal morphology, dependent upon the factor of biological sex. A centrifugal branch order analysis revealed no significant alterations in dendritic branching between male HIV-1 Tg and male control animals **(A)**. However, female HIV-1 Tg animals exhibited an increased relative frequency of dendrites on lower order branches relative to female control animals **(B)**. Assessment of neuronal arbor complexity utilized a Sholl analysis, revealing similar findings. Specifically, there were no significant differences in neuronal arbor complexity between male HIV-1 Tg and male control animals **(C)**. However, female HIV-1 Tg animals showed a decreased number of intersections on radii closer to the soma relative to control females **(D)**.

Complementary analyses were conducted to determine the locus of the interaction. In male animals, no significant differences in branch order distribution were observed between HIV-1 Tg and control animals (Figure 2A; *p* > 0.05). However, female HIV-1 Tg animals exhibited a profound shift in the distribution of dendritic branches, with an increased relative frequency of lower order branches relative to female control animals (Figure 2B). A generalized linear mixed effects model conducted on the number of dendrites at each branch order in female animals confirmed these observations, revealing a significant branch order × genotype interaction (*F*_(1,548)_ = 35.33, *p* ≤ 0.001).

#### Sholl Analysis

Sholl analyses were used to compare neuronal arbor complexity between control and HIV-1 Tg rats by quantifying the number of dendritic intersections occurring every 10 μm from the soma. There was no significant radius × genotype interaction or radius × sex interaction (*p* > 0.05). However, presence of the HIV-1 transgene had a profound effect on neuronal arbor complexity in MSNs of the NAc core subregion, dependent upon the factor of biological sex, evidenced by a significant radius × sex × genotype interaction (*F*_(1,2351)_ = 9.7, *p* ≤ 0.002).

Complementary analyses were conducted to determine the locus of the interaction. There was no significant difference in neuronal arbor complexity between HIV-1 Tg males and control males (Figure 2C; *p* > 0.05). However, female HIV-1 Tg animals showed a decreased number of intersections on radii closer to the soma relative to control females (Figure 2D). A mixed-design ANOVA conducted on the number of radii intersections in female animals confirmed these observations, revealing a significant radius × genotype interaction (*F*_(1,1186)_ = 8.5, *p* ≤ 0.004).

Notably, neither presence of the HIV-1 transgene nor the factor of biological sex influenced the total dendrite length (*p* > 0.05). Thus, HIV-1 Tg animals exhibited sex dependent alterations in branch order and Sholl analyses, suggestive of an alteration in neuronal morphology independent of alterations in total dendritic length.

### Examination of Dendritic Spine Morphology, Assessed Using Dendritic Spine Volume, Revealed Profound Alterations in Male, but Not Female, HIV-1 Tg Animals

**Figure 3:**
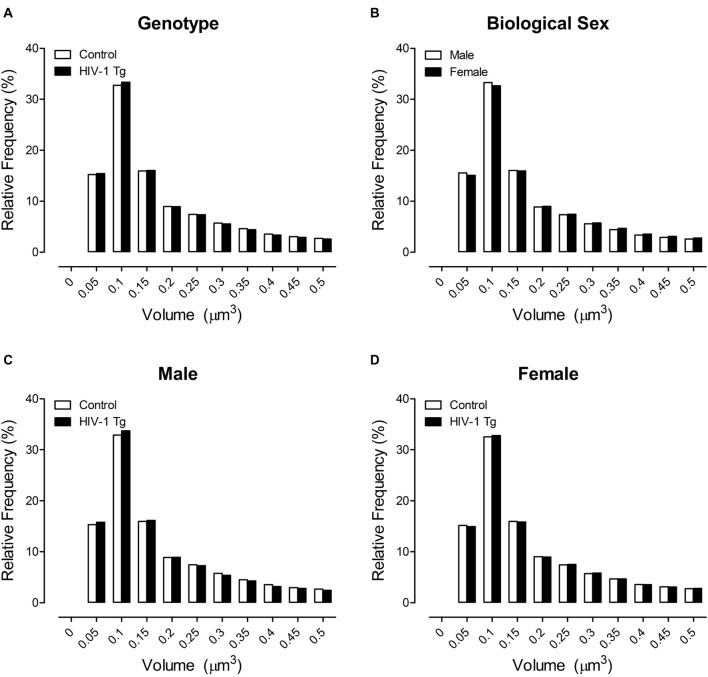
Across a population of 500,000+ spines, a shift in the distribution of dendritic spine volume was observed dependent upon the factor of biological sex **(B)**, but not genotype **(A)**. More critically, however, presence of the HIV-1 transgene had a profound influence on the distribution of dendritic spine volume dependent upon the factor of biological sex. Male HIV-1 Tg animals displayed a distribution shift with an increased relative frequency of spines with lower volume relative to male control animals **(C)**; an effect not observed in female HIV-1 Tg animals **(D)**.

The primary morphological parameter assessed in the present study was dendritic spine volume (Figure 3), a parameter that encapsulates both dendritic spine length and dendritic spine head diameter. No significant population shift in the distribution of dendritic spine volume was observed in HIV-1 Tg and control animals (Figure 3A; *p* > 0.05). However, the factor of biological sex (Figure 3B; Sex × Bin Interaction: *F*_(1,1201)_ = 26.4, *p* ≤ 0.001) significantly influenced the distribution of dendritic spine volume. Specifically, female animals, independent of genotype, displayed a rightward shift with an increased relative frequency of dendritic spines with increased volume, relative to male animals.

More critically, however, presence of the HIV-1 transgene, had a profound influence on the distribution of dendritic spine volume dependent upon the factor of biological sex (Figures 3C,D; Bin × Genotype × Sex Interaction: (*F*_(1,1201)_ = 5.8, *p* ≤ 0.020). Complementary analyses were conducted to determine the locus of the interaction. Male HIV-1 Tg animals showed a shift in the distribution of dendritic spine volume, with an increased relative frequency of dendritic spines with decreased volume, relative to male control animals (Figure 3C). A generalized linear mixed effects model conducted on the number of dendritic spines within each bin in male animals confirmed these observations, revealing a significant bin × genotype interaction (*F*_(1,595)_ = 7.1, *p* ≤ 0.001). In sharp contrast, there was no significant distributional shift in dendritic spine volume observed in female HIV-1 Tg animals relative to female control animals (Figure 3D; *p* > 0.05).

### Dendritic Spine Alterations Explain Significant Genotypic Variance, Dependent Upon Biological Sex

**Figure 4:**
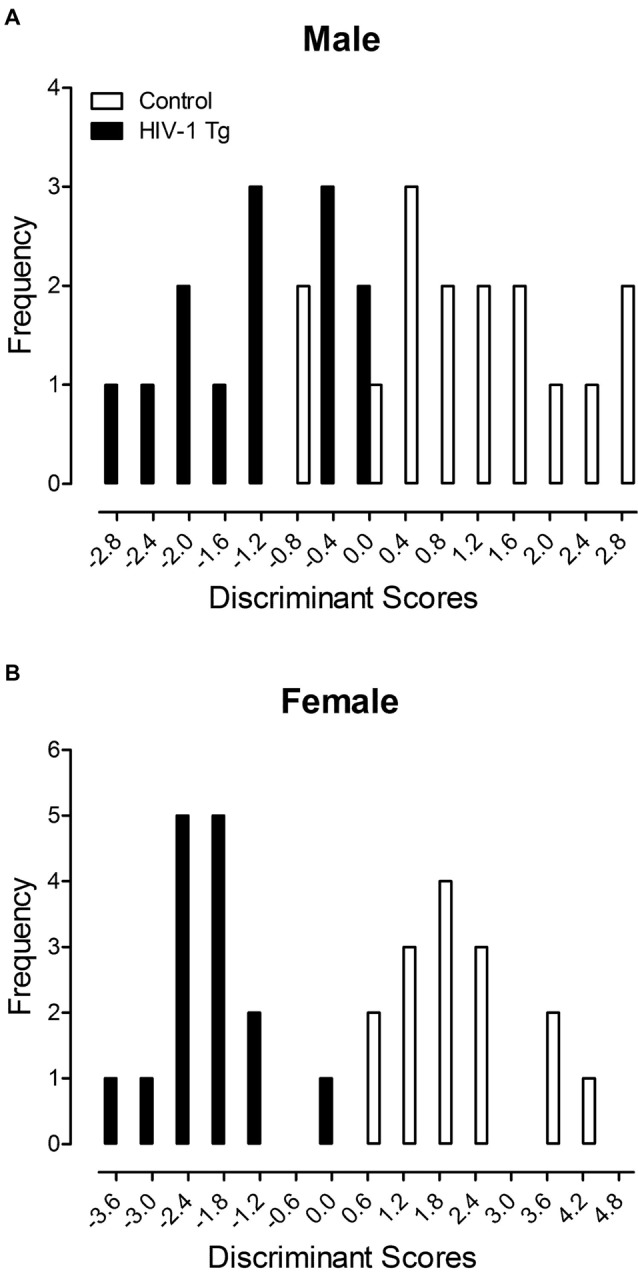
A discriminant function analysis (DFA) was utilized to determine whether dendritic spine measurements could classify animals based on genotype and biological sex. Male animals **(A)** were correctly classified for presence of the HIV-1 transgene (jack-knifed classification) with 80.0% accuracy using assessments of dendritic spine connectivity and dendritic spine volume. Female animals **(B)** were correctly classified for presence of the HIV-1 transgene (jack-knifed classification) with 90% accuracy utilizing measurements of dendritic spine connectivity, dendritic branching and neuronal arbor complexity.

A DFA was utilized to determine whether dendritic spine measurements could classify animals based on genotype and biological sex (Figure 4). Variables input into the stepwise DFA were chosen based on significant results reported above. Specifically, the analysis of male animals included variables for dendritic spine connectivity and dendritic spine volume, whereas the analysis of female animals included variables for dendritic spine connectivity, dendritic branching and neuronal arbor complexity.

Male HIV-1 Tg and male control animals (Figure 4A) were maximally separated (canonical correlation of 0.76) by selecting five variables corresponding to dendritic spine connectivity (i.e., Relative Frequency of Dendritic Spines at 20 μm, 130 μm and 190 μm) and dendritic spine volume (i.e., Relative Frequency of Dendritic Spines within the 0.1 μm^3^ and 0.25 μm^3^ bin). Male animals were correctly classified for presence of the HIV-1 transgene (jack-knifed classification) with 80.0% accuracy (Approximation of Wilks’ λ of 0.426, $\chi_{\left(5\right)}^{2}$ = 21.8, *p* ≤ 0.001), explaining 64% of the genotypic variance in male animals.

Female HIV-1 Tg and female control animals (Figure 4B) were maximally separated (canonical correlation of 0.91) by selecting 10 variables corresponding to dendritic spine connectivity (i.e., Relative Frequency of Dendritic Spines at 20 μm, 60 μm, 80 μm, 140 μm and 170 μm), dendritic branching complexity (i.e., Number of Branches at Branch Order 1) and neuronal arbor complexity (i.e., Number of Intersections at 60 μm, 80 μm, 100 μm and 160 μm). Female animals were correctly classified for presence of the HIV-1 transgene (jack-knifed classification) with 90.0% accuracy (Approximation of Wilks’ λ of 0.178, $\chi_{\left(10\right)}^{2}$ = 39.7, *p* ≤ 0.001), explaining 81.0% of the genotypic variance in female animals.

### HIV-1 Tg Rats Exhibit Intact Sensory and Motor System Function, Supporting the Functional Health of the HIV-1 Tg Rat at an Advanced Age

#### Cross-Modal PPI

Sensory system function, including auditory and visual system function, can be readily assessed using PPI of the ASR (Ison, 1984; Wecker et al., 1985; Crofton and Sheets, 1989). Amplitude of the ASR can be utilized to assess the ability of the subject (i.e., rat, mouse, human) to detect the discrete prestimulus (Wecker et al., 1985).

**Figure 5:**
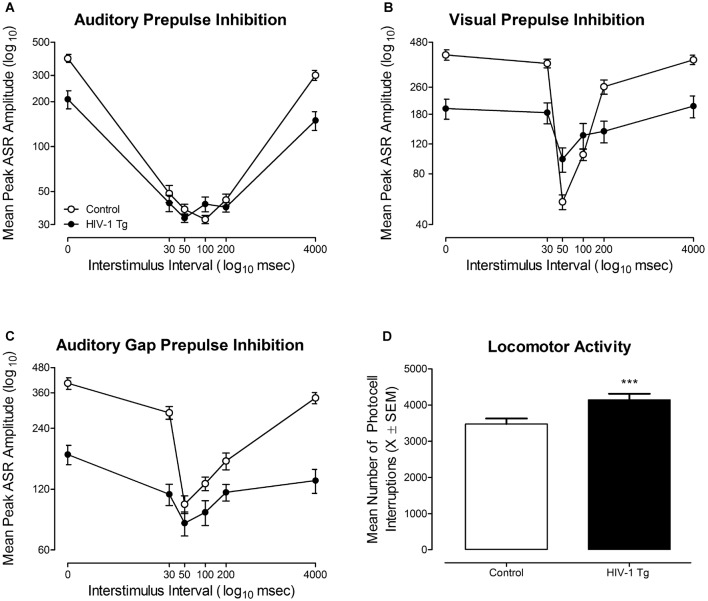
The functional health of the HIV-1 Tg rat, assessed using cross-modal prepulse inhibition (PPI), gap prepulse inhibition (gap-PPI) and locomotor activity, is illustrated.** (A)** Mean peak auditory startle response (ASR) amplitude (±SEM) for auditory PPI is illustrated as a function of genotype (HIV-1 Tg vs. Control). Robust inhibition to the presentation of an auditory prestimulus was observed, supporting the integrity of auditory system function at 18 months of age. **(B)** Mean peak ASR amplitude (±SEM) for visual PPI is illustrated as a function of genotype (HIV-1 Tg vs. Control). Both HIV-1 Tg and control animals displayed robust inhibition to the presentation of a visual prestimulus, supporting the integrity of visual system function at 18 months of age. **(C)** Mean peak ASR amplitude (±SEM) for auditory gap-PPI is illustrated as a function of genotype (HIV-1 Tg vs. Control). Robust inhibition to the absence of background noise supports the generality of auditory system function at 18 months of age. **(D)** Mean number of photocell interruptions across the 60-min test session are illustrated as a function of genotype (HIV-1 Tg, control; ±SEM). Both HIV-1 Tg and control animals displayed significant motor activity throughout the 60 min test session, supporting the integrity of gross-motoric system function. HIV-1 Tg animals exhibited a significantly greater mean number of photocell interruptions relative to control animals. ***p ≤ 0.001.

In auditory PPI (Figure 5A), HIV-1 Tg and control animals displayed robust inhibition to the presentation of an auditory prestimulus, supporting the integrity of auditory system function at 18 months of age. Control rats displayed maximal peak inhibition at the 100 ms ISI, whereas, HIV-1 Tg rats displayed maximal peak inhibition at the 50 ms ISI. HIV-1 Tg animals also displayed a relative insensitivity to the manipulation of ISI, relative to controls, evidenced by a flatter ISI function. The mixed-model ANOVA conducted on the mean peak ASR amplitudes for auditory PPI revealed a significant genotype × ISI interaction (*F*_(5,290)_ = 32.1, *p* ≤ 0.001) and an ISI × sex interaction (*F*_(5,290)_ = 5.6, *p* ≤ 0.001). Main effects of genotype (*F*_(1,58)_ = 23.9, *p* ≤ 0.001), sex (*F*_(1,58)_ = 6.8, *p* ≤ 0.011), and ISI (*F*_(5,290)_ = 228.1, *p* ≤ 0.001) were also observed.

In visual PPI (Figure 5B), robust inhibition to the presentation of a visual prestimulus was observed at the 50 ms ISI in both HIV-1 Tg and control animals, supporting the integrity of the visual system at 18 months of age. Additionally, HIV-1 Tg rats displayed a relative insensitivity to the manipulation of ISI, evidenced by a flatter ISI function, relative to control animals. Observations in visual PPI were confirmed using a mixed-design ANOVA, revealing a significant genotype × ISI interaction (*F*_(5,290)_ = 36.5, *p* ≤ 0.001). Main effects of genotype (*F*_(1,58)_ = 17.0, *p* ≤ 0.001), sex (*F*_(1,58)_ = 4.9, *p* ≤ 0.032), and ISI (*F*_(5,290)_ = 104.9, *p* ≤ 0.001) were also observed. Thus, the assessment of cross-modal PPI confirmed the integrity of sensory system function at an advanced age, extending previous reports (McLaurin et al., 2018a) to 18 months of age.

#### Gap-PPI

In gap PPI (Figure 5C), a 20 ms absence of background (i.e., gap) served as a prestimulus, relative to the presentation of an added prestimulus in cross-modal PPI. HIV-1 Tg and control animals displayed robust inhibition to the absence of background noise, providing evidence for the generality of auditory system function. Maximal inhibition was observed at the 50 ms ISI in both HIV-1 Tg and control animals. However, HIV-1 Tg animals, once again, displayed a relative insensitivity to the manipulation of ISI, evidenced by a relatively flatter ISI function relative to controls. Observations were confirmed using a mixed-design ANOVA, which revealed a significant genotype × ISI interaction (*F*_(5,290)_ = 52.8, *p* ≤ 0.001) and a significant ISI × sex interaction (*F*_(5,290)_ = 3.06, *p* ≤ 0.010). Main effects of genotype (*F*_(1,58)_ = 40.7, *p* ≤ 0.001) and ISI (*F*_(5,290)_ = 138.4, *p* ≤ 0.001) were also noted. Assessment of gap-PPI supports the generality of sensory system integrity in the HIV-1 Tg rat at 18 months of age.

#### Locomotor Activity

The integrity of gross-motoric system function at an advanced age, illustrated in Figure 5D, was assessed using the mean number of photocell interruptions within a test session (i.e., 60 min) as an index of motor behavior (Pierce and Kalivas, 2007). At 18 months of age, both HIV-1 Tg and control animals exhibited significant motor activity across the 60 min test session. HIV-1 Tg animals displayed an increased number of total motor movements (Mean Total Photocell Interruptions: 4145.3 ± 167.6) relative to controls (Mean Total Photocell Interruptions: 3476.1 ± 152.5) across the test session. The overall ANOVA confirmed these observations revealing a significant main effect of genotype (*F*_(1,58)_ = 11.8, *p* ≤ 0.001) and a main effect of sex (*F*_(1,58)_ = 15.9, *p* ≤ 0.001). Thus, the assessment of locomotor activity confirmed the integrity of gross-motoric system function at an advanced age, extending previous reports (McLaurin et al., 2018a) to 18 months of age.

## Discussion

Profound alterations in synaptic connectivity were observed in MSNs of the NAc core subregion in the population of HIV-1 Tg animals sampled. The functional health of the HIV-1 Tg rat, assessed via cross-modal PPI, gap-PPI and locomotor activity, reveals the integrity of sensory and motor system function at an advanced age (i.e., 18 months of age). HIV-1 Tg animals, relative to controls, displayed a prominent shift in the distribution of dendritic spines, independent of biological sex, supporting an alteration in synaptic connectivity. Specifically, HIV-1 Tg animals exhibited an increased relative frequency of thin and mushroom spines on more proximal branches, whereas stubby spines were prevalent on more distal branches. The mechanism underlying alterations in synaptic connectivity, however, is likely dependent upon biological sex. Alterations in dendritic branching and neuronal arbor complexity were observed in HIV-1 Tg females, but not HIV-1 Tg males, relative to controls, supporting an alteration in neurotransmission. A leftward distributional shift in dendritic spine volume was evidenced in HIV-1 Tg males, but not HIV-1 Tg females, relative to controls, suggesting decreased synaptic area. Dendritic spine alterations accounted for significant genotypic variance, dependent upon biological sex, supporting a potential underlying neural mechanism for HIV-1 associated apathy. Thus, sex-dependent synaptic dysfunction in MSNs of the NAc core subregion supports a key structural loci for apathy in the HIV-1 Tg rat and heralds the development of sex-based therapeutics and cure strategies.

Classic dendritic spine nomenclature divides spines into three primary categories (i.e., thin, stubby, mushroom; Peters and Kaiserman-Abramof, 1970) dependent upon morphological characteristics. Thin spines, the predominant spine type in both HIV-1 Tg and control animals, independent of biological sex, are characterized by a long, thin neck and a small bulbous head. Mushroom spines are commonly associated with greater stability, containing a high head volume to neck volume ratio. Stubby spines, in sharp contrast, are devoid of a spine neck (Jones and Powell, 1969; Peters and Kaiserman-Abramof, 1970) and have an approximately equal head and neck volume ratio. Dendritic spines, which are reflective of functionality and capacity for structural change (Lai and Ip, 2013), serve as the main postsynaptic compartment of excitatory synapses (Spiga et al., 2014). Typically, excitatory synapses occur on dendritic spine heads, with some spines receiving additional input on their neck (Yuste, 2010). Morphological changes in dendritic spines, therefore, may underlie alterations in synaptic connectivity.

Within the NAc, MSNs receive afferent projections from multiple brain regions, playing a central role in the integration of cortical and thalamic input (Spiga et al., 2014). Specifically, the soma and more proximal dendrites are primarily innervated by afferents from other MSNs (Groves, 1983). More distal dendrites, however, receive glutamatergic afferents from the PFC and dopaminergic afferents from the VTA (Spiga et al., 2014); innervation which leads to dual synapses on a single dendritic spine forming a “synaptic triad” (Freund et al., 1984). Within the “synaptic triad,” glutamatergic afferents establish synaptic contact on the dendritic spine head, while dopaminergic afferents are targeted at the dendritic spine neck (Freund et al., 1984). Most notably, in MSNs, the dendritic spine neck receives approximately 70% of dopaminergic synapses (Zahm, 1992). Although the precise role of the “synaptic triad” is unclear (Koos et al., 2011), it seems to suggest that even modest alterations in dendritic spines may have an effect on the entire neural circuitry (Spiga et al., 2014).

**Figure 6:**
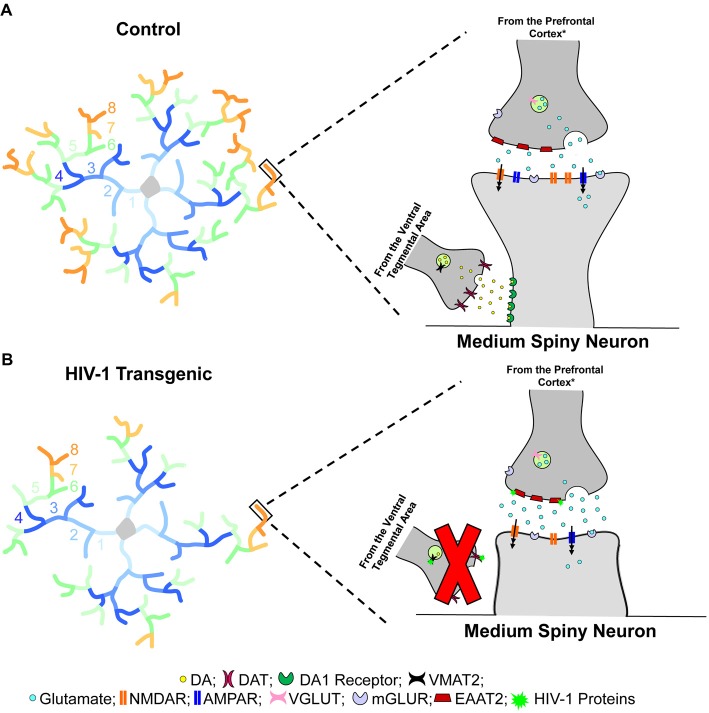
Dendritic spine connectivity in medium spiny neurons (MSNs) is illustrated as a function of genotype (HIV-1 Tg vs. Control). Dendritic branching complexity is illustrated for branch orders 1 through 8. Control animals **(A)** exhibited complex dendritic branching and an increased relative frequency of mushroom spines on more distal branches. Mushroom spines, commonly associated with greater stability, contain a high head volume to neck volume ratio (Jones and Powell, 1969; Peters and Kaiserman-Abramof, 1970). In sharp contrast, HIV-1 Tg animals **(B)** displayed decreased dendritic branch complexity, dependent upon biological sex, with an increased relative frequency of stubby spines on more distal branches. Stubby spines are morphologically characterized by the absence of a dendritic spine neck and an approximately equal head and neck volume ratio (Jones and Powell, 1969; Peters and Kaiserman-Abramof, 1970). Spines located on the more distal dendrites receive glutamatergic afferents from the prefrontal cortex (PFC)* and dopaminergic afferents from the ventral tegmental area (VTA; Spiga et al., 2014); innervation which leads to dual synapses on a single dendritic spine forming a “synaptic triad” (Freund et al., 1984). Most notably, the morphological characteristics of dendritic spines, serving as the main postsynaptic compartment of excitatory synapses (Spiga et al., 2014), reflect functionality and capacity for structural change (Lai and Ip, 2013). Specifically, the prevalence of mushroom spines, having a high head volume to neck volume ratio, on more distal branches in control animals suggests the formation of a “synaptic triad,” whereby glutamatergic afferents establish synaptic contact on the dendritic spine head, while dopaminergic afferents are targeted at the dendritic spine neck (Freund et al., 1984). In sharp contrast, the increased prevalence of stubby spines on more distal branches in HIV-1 Tg animals suggests a failure to form the “synaptic triad.” Specifically, the absence of a dendritic spine neck, which receives approximately 70% of dopaminergic synapses (Zahm, 1992), prevents the dendritic spines from receiving dopaminergic afferents from the VTA, leading to alterations in neurotransmission commonly observed in HIV-1 (e.g., Moran et al., 2013b; Lee et al., 2014; Zhu et al., 2016; Javadi-Paydar et al., 2017; Sinharay et al., 2017; Bertrand et al., 2018; McLaurin et al., 2018b). *It is critical to note that the figure represents one potential neural mechanism underlying apathy in the HIV-1 Tg rat. In addition to the PFC, the thalamus and hippocampus (Harris and Stevens, 1989), as well as the amygdala (Bredt and Nicoll, 2003) also send glutamatergic inputs to MSNs of the nucleus accumbens (NAc).

HIV-1 Tg animals, independent of biological sex, displayed prominent alterations in the distribution of dendritic spines, supporting a profound alteration in synaptic connectivity. Specifically, HIV-1 Tg animals, relative to controls, exhibited a preponderance of thin and mushroom spines on the more proximal dendrites, receiving collaterals from other MSNs. However, in HIV-1 Tg animals, stubby spines were predominant on the more distal dendrites; an effect which was more pronounced in female HIV-1 Tg animals. The absence of a dendritic spine neck, a primary morphological characteristic of stubby spines, suggests that HIV-1 Tg animals failed to receive dopaminergic afferents from the VTA (Figure 6); an effect which may mechanistically underlie dopaminergic alterations commonly observed in the HIV-1 Tg rat (e.g., Lee et al., 2014; Zhu et al., 2016; Javadi-Paydar et al., 2017; Sinharay et al., 2017). Observations of profound alterations in synaptic connectivity may generalize to other brain regions, including the PFC following psychostimulant exposure (McLaurin et al., 2018b). Alterations in the distribution of dendritic spines in HIV-1 Tg animals, therefore, prevent the formation of a “synaptic triad,” which may underlie changes in circuitry connectivity (i.e., VTA-NAc-PFC). The precise neural mechanism underlying alterations in synaptic connectivity, however, is likely dependent upon the factor of biological sex.

Alterations in neuronal morphology, including dendritic branching and neuronal arbor complexity, were observed in female HIV-1 Tg animals, but not male HIV-1 Tg animals, relative to controls. The development and maintenance of the neuronal arbor is likely influenced by synaptic activity, although to date, there is no clear consensus on the precise role (Cline, 2001). One hypothesis, however, is that dendritic branching complexity may be influenced by glutamate receptor activity. Specifically, treatment with glutamate receptor antagonists leads to decreases in neuronal arbor growth, as well as synaptic transmission (Rajan and Cline, 1998; Haas et al., 2006). Notably, HIV-1 viral proteins, specifically Tat or gp120, lead to increased extracellular concentrations of glutamate, evidenced by an inhibition of glutamate uptake in cultured glial cells (Patton et al., 2000; Wang et al., 2003; Gupta et al., 2010; Melendez et al., 2016). Most critically, however, an *in vivo* study in gp120 Tg mice demonstrated a significant decrease in neuronal glutamate uptake in the striatum (Melendez et al., 2016). Thus, decreased dendritic complexity in female HIV-1 Tg animals may be due, at least in part, to alterations in glutamate neurotransmission.

Morphologically, male HIV-1 Tg animals, but not female HIV-1 Tg animals, displayed a population shift towards dendritic spines with decreased volume relative to controls. The morphological heterogeneity of dendritic spine shape likely reflects the functional features of synapses (Yuste, 2010). In regards to dendritic spine volume, strong correlations (i.e., *r* ≥ 0.83) between spine volume and synaptic area have been previously reported (Freire, 1978; Arellano et al., 2007). Furthermore, increased synaptic area has been correlated with both the number of presynaptic vesicles (Harris and Stevens, 1988), as well as the number of docked vesicles (Schikorski and Stevens, 1999), which is, in turn, associated with the likelihood of neurotransmitter release (Rosenmund and Stevens, 1996; Schikorski and Stevens, 2001). Additionally, increased synaptic area has also been correlated with an increased number of postsynaptic receptors (Yuste, 2010). Collectively, decreased dendritic spine volume suggests that HIV-1 Tg male animals have decreased synaptic area, fewer docked vesicles, fewer postsynaptic receptors and, thus, altered neurotransmitter release; findings which have been reported following HIV-1 viral protein exposure (e.g., Kim et al., 2008; Gelman et al., 2012; Stevens et al., 2015).

Neural circuitry alterations, resulting from neurotransmitter alterations and synaptic dysfunction, may mechanistically underlie HIV-1 associated apathy in the post-cART era. Specifically, in the present study, dendritic spine alterations in MSNs of the NAc core subregion explain significant genotypic variance (i.e., 64%–81%), dependent upon biological sex. Mechanistically, we have previously reported a prominent relationship between neurochemical alterations in the DA transporter (DAT) and motivational dysregulation in the HIV-1 Tg rat (Bertrand et al., 2018). Thus, synaptic dysfunction, leading to neural circuitry alterations, supports a primary mechanism for HIV-1 associated apathy in the post-cART era.

The importance of the systematic investigation of sex differences in neural circuitry alterations, as in the present article, cannot be understated. Women represent approximately half of the individuals living with HIV-1 (UNAIDS, 2016), yet remain underrepresented in both clinical and preclinical studies (Maki and Martin-Thormeyer, 2009; Maki et al., 2015). More recent clinical studies have systematically evaluated sex differences in HIV-1 associated neurocognitive disorders, revealing greater neurocognitive impairment in HIV-1 seropositive women relative to HIV-1 seropositive men (Royal et al., 2016; Maki et al., 2018); results which were translationally modeled in the HIV-1 Tg rat (e.g., McLaurin et al., 2016, 2017; Rowson et al., 2016). To date, however, no clinical or preclinical study has systematically evaluated sex differences in HIV-1 associated apathy. Prominent sex differences in the present study, however, highlights this critical need. Additionally, further studies are needed to directly assess whether sex differences in the HIV-1 Tg rat represent alterations in disease progression (e.g., McLaurin et al., 2018a) or, more simply, sex differences in circuitry function.

The HIV-1 Tg rat serves as a valid and reliable animal system to translationally model the effect of long-term HIV-1 viral protein expression in the post-cART era on neurocognitive function (Vigorito et al., 2015) and HIV-1 associated apathy (Bertrand et al., 2018). The deletion of the viral proteins -gag and -pol renders the HIV-1 Tg rat non-infectious, however, the remaining 7 HIV-1 viral proteins are expressed constitutively throughout the lifespan (Peng et al., 2010; Abbondanzo and Chang, 2014). Notably, the assessment of cross-modal PPI, gap-PPI and locomotor activity provides an experimental paradigm to assess the functional health of the HIV-1 Tg rat at an advanced age. Robust inhibition to the presence of either an auditory or visual prestimulus, as in cross-modal PPI, and the absence of background noise in gap-PPI, provides strong evidence for intact sensory (i.e., auditory, visual) system function, extending our previous reports (McLaurin et al., 2018a), to 18 months of age. Additionally, locomotor activity demonstrated the integrity of gross-motoric system function at 18 months of age. Thus, in combination with previous reports through 16 months of age (McLaurin et al., 2018a), the present data supports the functional health of the HIV-1 Tg rat throughout the majority of the animal’s functional lifespan.

It is critical to note that the proposed circuitry alterations underlying apathy in the HIV-1 Tg rat are not exclusionary. Specifically, in addition to the PFC, the thalamus and hippocampus (Harris and Stevens, 1989), as well as the amygdala (Bredt and Nicoll, 2003), also send glutamatergic inputs to MSNs of the NAc. For example, HIV-1 viral proteins (i.e., gp120) significantly enhanced NMDAR-mediated EPSC in rat hippocampus slices, which may subsequently alter excitatory synaptic transmission mediated by glutamate (Zhou et al., 2017). Thus, it is possible that alterations in glutamate neurotransmission from multiple brain regions influence neuronal morphology in the MSNs of the NAc. However, the focus on frontal-subcortical circuit dysfunction as a neural basis of HIV-1 associated apathy is supported by clinical (e.g., Cole et al., 2007) and preclinical (e.g., Vigorito et al., 2007; Moran et al., 2014; McLaurin et al., 2017) studies more broadly establishing frontal-subcortical circuitry dysfunction in HIV-1.

Collectively, long-term HIV-1 viral protein exposure leads to profound synaptic dysfunction, evidenced by alterations in synaptic connectivity, in the HIV-1 Tg rat. Prominent alterations in the distribution of dendritic spines, independent of biological sex, supports an alteration in synaptic connectivity. Further, sex differences in neuronal morphology and dendritic spine volume suggest that the circuitry underlying synaptic connectivity alterations is sex dependent. Overall, results suggest that the HIV-1 Tg rat fails to form a “synaptic triad” at the distal branches, which may lead to neurotransmitter system alterations and circuitry dysfunction commonly observed in the HIV-1 Tg rat. Thus, synaptic dysfunction in MSNs of the NAc core subregion supports a key, sex-dependent, structural loci for apathy in the HIV-1 Tg rat and heralds a target for the development of sex-based therapeutics and cure strategies.

## Author Contributions

RB and CM conceived and designed the experiments. KM, HL, CM and RB performed the experiments. KM, AL and CM analyzed the data. KM, AC, CM and RB: wrote the article. KM, AC, HL, AL, CM and RB critical appraisal and approval of final manuscript.

## Conflict of Interest Statement

The authors declare that the research was conducted in the absence of any commercial or financial relationships that could be construed as a potential conflict of interest.

## Supplementary Material

FIGURE S1 The distribution of dendritic spines on successive radii is illustrated as a function of spine type (i.e., Thin Spines **(A,B)**, Mushroom Spines **(C,D)**, and Stubby Spines **(E,F)**) and biological sex (i.e., Male **(A,C,E)**, Female **(B,D,F)**). HIV-1 Tg animals displayed an increased relative frequency of thin and mushroom spines on more proximal branches relative to control animals; the magnitude of which was dependent upon biological sex. In sharp contrast, HIV-1 Tg animals displayed an increased relative frequency of stubby spine on more distal branches relative to control animals; an effect which was not influenced by the factor of biological sex.
